# Beyond BMI: cardiometabolic measures as predictors of impulsivity and white matter changes in adolescents

**DOI:** 10.1007/s00429-023-02615-0

**Published:** 2023-02-13

**Authors:** Anna Prunell-Castañé, María Ángeles Jurado, Jonatan Ottino-González, Xavier Prats-Soteras, Consuelo Sánchez Garre, Neus Cano Marco, Paloma Salas Gómez-Pablos, Isabel García-García, Maite Garolera

**Affiliations:** 1 Departament de Psicologia Clínica i Psicobiologia, Facultat de Psicologia, Universitat de Barcelona, Passeig de la Vall d’Hebron, 171, 08035 Barcelona, Spain; 2 Institut de Neurociències, Universitat de Barcelona, Barcelona, Spain; 3 Institut de Recerca Sant Joan de Déu, Barcelona, Spain; 4 Department of Endocrinology, Children’s Hospital Los Angeles, Los Angeles, USA; 5 Pediatric Endocrinology Unit, Hospital de Terrassa, Consorci Sanitari de Terrassa, Barcelona, Spain; 6 Brain, Cognition and Behavior: Clinical Research, Consorci Sanitari de Terrassa, Barcelona, Spain; 7 Catlab, Consorci Sanitari de Terrassa, Barcelona, Spain; 8 Neuropsychology Unit, Hospital de Terrassa, Consorci Sanitari de Terrassa, Barcelona, Spain

**Keywords:** Impulsivity, Adolescence, Obesity, White matter, Cardiometabolic

## Abstract

Obesity is characterized by cardiometabolic and neurocognitive changes. However, how these two factors relate to each other in this population is unknown. We tested the association that cardiometabolic measures may have with impulse behaviors and white matter microstructure in adolescents with and without an excess weight. One hundred and eight adolescents (43 normal-weight and 65 overweight/obesity; 11–19 years old) were medically and psychologically (Temperament Character Inventory Revised, Three-Factor Eating Questionnaire-R18, Conners’ Continuous Performance Test-II, Stroop Color and Word Test, Wisconsin Card Sorting Test, Kirby Delay Discounting Task) evaluated. A subsample of participants (*n* = 56) underwent a brain magnetic resonance imaging acquisition. In adolescents, higher triglycerides and having a body mass index indicative of overweight/obesity predicted a more impulsive performance in Conners’ Continuous Performance Test-II (higher commission errors). In addition, higher glucose and diastolic blood pressure values predicted increments in the Three-Factor Eating Questionnaire-R18 emotional eating scale. Neuroanatomically, cingulum fractional anisotropy showed a negative relationship with glycated hemoglobin. The evaluation of the neurocognitive differences associated with obesity, usually based on body mass index, should be complemented with cardiometabolic measures.

**Supplementary Information:**

The online version contains supplementary material available at 10.1007/s00429-023-02615-0.

## Introduction

Overweight and obesity represent a major public health concern. Since 1975, the prevalence of excess weight among children and adolescents has more than quadrupled. Psychological, biological, and sociocultural factors can contribute to the development of overweight/obesity (World Health Organization 2020). Impulsivity is a multidimensional construct that can correlate with the expression of excess weight, since it may lead to a rapid and unplanned reaction towards food. Impulsivity involves urgency, lack of perseverance and premeditation, and sensation seeking (Mobbs et al. 2010). Importantly, it is a broad concept that has diverse traits and manifestations. A systematic review (Liang et al. 2014) highlighted that, although the literature had mixed results, most studies found more pronounced impulsive behaviors in children and adolescents with overweight/obesity.

Overweight/obesity is also associated with neuroanatomic changes. White matter (WM) differences in this population have been studied with diffusion tensor imaging (DTI). DTI research evidenced WM alterations related to excess weight. Two common measures of WM microstructure are fractional anisotropy (FA) and mean diffusivity (MD). Lower FA and higher MD may reflect disturbances in WM microstructure. In adults, many studies found a negative association between body mass index (BMI) and FA in several WM tracts (Verstynen et al. 2012; Xu et al. 2013; Papageorgiou et al. 2017; Rodrigue et al. 2019). A positive association between BMI and MD was also described (Xu et al. 2013); although another study did not find associations between BMI and MD (Papageorgiou et al. 2017). In children and adolescents, there are mixed results regarding BMI and WM microstructure. There was described a positive (Ou et al. 2015), negative (Alarcón et al. 2016), and no relationship (Alosco et al. 2014) between BMI and FA. Concerning BMI and MD, no significant relationship was found in two studies (Ou et al. 2015; Alarcón et al. 2016), while another observed higher MD values in participants with excess weight (Carbine et al. 2019).

Moreover, not only BMI is related to WM microstructure. Obesity is usually accompanied by cardiometabolic changes that might have a potential impact on neural integrity. How WM integrity is negatively related to cardiometabolic measures has been studied under a broader approach: metabolic syndrome. Metabolic syndrome has been related to WM microstructure (Segura et al. 2009) and macrostructure (Morys et al. 2021), and in adolescents (Yau et al. 2012) and adults (Segura et al. 2009). Studies evaluating the independent effect that each cardiometabolic variable may have on WM are sparse and focused on adults (Verstynen et al. 2013; Lou et al. 2014; Cox et al. 2019; Johnson et al. 2019).

While BMI is a commonly used indirect measure of overweight/obesity, it is an incomplete diagnostic tool (Barlow 2007).

Thus, to favor an integrative assessment of overweight/obesity, we complemented BMI with cardiometabolic variables. Despite the emerging interest in the study of cardiometabolic profile as a possible biomarker of impulsivity—especially focused on mental health (Conklin and Stanford 2008; Kavoor et al. 2017; Messaoud et al. 2017)—, the relationship of cardiometabolic measures with impulsivity and neuroanatomical differences in adolescents with overweight/obesity remains unknown.

The present study evaluates, in adolescents with normal weight and overweight/obesity, the association of cardiometabolic risk factors with (1) impulsive behaviors and (2) WM microstructure. We hypothesize that greater cardiometabolic risk factors might be related to (1) more impulsive behaviors, and (2) to WM microstructure differences. Specifically, we expect to find lower FA and higher MD values in association with greater cardiometabolic risk.

## Materials and methods

### Participants

We recruited 108 adolescents (mean age = 15 ± 2.02 years old) from public primary care centers. From this sample, 53 participants were already included in a previous work regarding inflammation and grey matter (Prats-Soteras et al. 2020). Inclusion criteria involved being from 11 to 19 years old and having a BMI indicative of normal weight, overweight or obesity. Participants were classified into two groups: normal-weight (*n* = 43) and overweight/obesity (*n* = 65). For that purpose, Cole and Lobstein centile curves (Cole and Lobstein 2012), that provide age and sex-specific cut-off points from 2 to 17 years old, were used to classify underage participants. In participants aged 18 and 19, according to the World Health Organization’s classification (World Health Organization 2020), those with a BMI between ≥ 18.5 and < 25 kg/m^2^ were classified as normal-weight, and those with BMI ≥ 25 kg/m^2^ were classified as overweight/obesity.

Exclusion criteria were (1) being prepubescent and (2) having a psychiatric, neurological, developmental, or systemic diagnosis. Participants did not take any chronic medication. Participants aged 18 and 19 were excluded if they met metabolic syndrome criteria (Alberti et al. 2009). For underage participants, and given the lack of general consensus to define pediatric metabolic syndrome (Yau et al. 2012), we used the cut-off points available from the Expert Panel on Integrated Guidelines for Cardiovascular Health and Risk Reduction in Children and Adolescents (De Jesus 2011) as exclusion criteria. Finally, participants showing global cognitive impairment (i.e., scalar score < 7 in the Weschler Adults Intelligence Scale-III/Weschler Intelligence Scale for Children-IV vocabulary subtest (WAIS-III/WISC-IV)), significant anxiety and depression symptoms (i.e., anxiety or depression symptoms total score ≥ 11 in the Hospital Anxiety and Depression Scale), and binge eating behaviors (i.e., score ≥ 20 in the Bulimia Inventory Test of Edinburgh) were also excluded.

This study was approved by the Institutional Ethics Committee. The research was conducted in accordance with the Helsinki Declaration. Written informed consent was obtained from all participants, or their respective legal guardian in underage participants, prior to entry into the study.

### Procedure

Participants were randomly contacted by phone and briefly interviewed about general health aspects. The first day, potential candidates were cited to undergo a complete medical evaluation and a fasting blood sample extraction in the Pediatric Endocrinology Unit at a Public Hospital. Subjects not presenting any medical comorbidity were neuropsychologically evaluated in the next days. Participants without claustrophobia or metal prothesis also underwent a brain magnetic resonance imaging (MRI) acquisition on a 3T MAGNETON Trio (Siemens, Germany) at a Public Hospital.

### Measures

#### Anthropometric and cardiometabolic measures

All anthropometric measurements were taken by a trained nurse from participants in light clothing without shoes: waist circumference, height (Holtan Limited Harpenden Stadiometer), weight (Seca 704 s) and BMI (kg/m^2^), which was transformed into BMI *z*-score. Pubertal stage was determined according to the Tanner scale of sexual maturity. Cardiometabolic measures included total cholesterol, high-density lipoprotein cholesterol (HDL-c), low-density lipoprotein cholesterol (LDL-c), triglycerides, glucose and glycated hemoglobin. Diastolic (DBP) and systolic blood pressure were manually determined twice (Riester Big Ben Round), and the mean of both determinations was used for posterior analyses. A description of cardiometabolic measures can be found in Supplementary Material-1a.

#### Impulsivity measures

The neuropsychological evaluation included: Temperament Character Inventory Revised (TCI-R), Three-Factor Eating Questionnaire-R18 (TFEQ-R18), Conners’ Continuous Performance Test II (CPT-II), Stroop Color and Word Test, Wisconsin Card Sorting Test (WCST) and Kirby Delay Discounting Task (DDT). We used the following scores to characterize impulsivity: higher scores in the TCI-R novelty seeking subscale, TFEQ-R18 uncontrolled and emotional eating scales, CPT-II commissions errors, WCST perseverative errors and DDT geometric mean; and lower scores in Stroop Interference score. A description of the neuropsychological assessment can be found in Supplementary Material-1b.

#### Image acquisition and diffusion-tensor imaging processing

DTI is a neuroimaging analysis technique that quantifies the directionality of water molecules in the brain (Basser et al. 1994). A common measure used in DTI studies is FA, which reflects the orientation dependence of water movement, and hence gives information about WM microstructure. FA measurement ranges from 0 to 1. Higher FA values may suggest well myelinated and undamaged tracts constraining the directional diffusion of water to be parallel, whereas lower FA values may reflect disturbed WM microstructure (Kullmann et al. 2015). Since FA is unspecific to the source driving the changes in WM microstructure, we tested a complementary diffusivity scalar. MD is the average of all eigenvalues with higher values meaning exacerbated cell permeability and thus WM impairments.

Fifty-six participants (25 normal-weight and 31 overweight/obesity) underwent an MRI. The parameters used to acquire the diffusion-weighted images are detailed in Supplementary Material-1c. Image processing was performed in FMRIB Software Library (FSL) v.6.0.4 and BrainSuite v.18a1. Estimated total intracranial volume was obtained for each participant using Freesurfer v.6.0 recon-all pipeline. An explanation of the imaging processing procedure can be found in Supplementary Material-1d.

Two approaches were considered in the DTI analysis: region of interest (ROI) and whole brain. Using the JHU ICBM-DTI-81 White-Matter Labels Atlas and JHU White Matter Tractography Atlas, five ROIs previously implicated in obesity and impulsivity were defined: cingulum, corona radiata, corpus callosum, inferior fronto-occipital fasciculus (IFOF) and internal capsule (Verstynen et al. 2012; Yau et al. 2012, 2014; Kullmann et al. 2015; Ou et al. 2015; Uhlmann et al. 2016; Jeong et al. 2017; Jiang et al. 2017; Papageorgiou et al. 2017; Bessette and Stevens 2019; Reich et al. 2019; Rodrigue et al. 2019; Carbine et al. 2019; Schreiner et al. 2020; Huang et al. 2020; Owens et al. 2020; Morys et al. 2021). FA and MD values of every ROI were averaged per individual and WM tract.

To explore additional relationships between the cardiometabolic profile and WM microstructure, whole-brain contrasts were implemented in FSL randomise with 10,000 iterations and a Threshold-Free Cluster Enhancement approach (Smith et al. 2006). Age, sex, BMI *z*-score and estimated total intracranial volume were modeled as nuisance variables. Due to the exploratory nature of these tests, a Bonferroni correction was applied. This method is more restrictive than the False Discovery Rate (FDR) correction used in the ROI analysis. Thus, for whole-brain analysis, statistical significance was set at *P* < 0.0015 (8 cardiometabolic variables × 2 WM measures × 2 contrasts).

### Data treatment and statistical analyses

Data manipulation and statistical procedures were performed in R statistical package v.4.0.5 and RStudio v.1.2.5033. Normality was determined with Shapiro–Wilk tests. Positively and non-normally distributed variables were transformed into their logarithmic form prior to any analyses. Briefly, we performed three different types of analyses: (a) Mean/median differences between BMI groups in all variables, (b) multiple regression: impulsivity or DTI measures ~ cardiometabolic variables + covariates, and (c) median differences of impulsivity measures between high/low cardiometabolic groups. Analysis (a) was performed to describe between-group differences. Analysis (b) was performed with all variables of interest, regardless of whether they were or not significantly different between groups in analysis (a). Analysis (c) was performed with only those variables that were significant in analysis (b).

Specifically, (a) independent sample *T*-tests, Mann–Whitney *U* tests and Chi-square tests were used to analyze between-group differences. Effect sizes were calculated using R packages *effsize* and *rcompanion*. Missing values for every variable were reported in Tables 1 and 2. We repeated these analyses for the subsample of participants that underwent an MRI acquisition (Supplementary Material, Tables S1 and S2). (b) Multiple regression analyses were performed to determine which cardiometabolic variables were the strongest predictors of impulsivity measures (covariates: age, sex, BMI *z*-score and intelligence quotient estimation (WAIS-III/WISC-IV vocabulary subtest)) and FA/MD differences (covariates: age, sex, BMI *z*-score and estimated total intracranial volume) in 5 WM tracts. Variance inflation factor (VIF) was used to assess multicollinearity within the predictors. To avoid a misestimation of the regression coefficients, total cholesterol was removed for having a VIF > 10. Confidence intervals at 95% for the regression coefficients were calculated as follows: [βi − 1.96 × SE(βi), βi + 1.96 × SE(βi)]. Multiple testing was controlled by FDR for 17 models (i.e., 7 impulsivity, 5 ROI-FA and 5 ROI-MD models). Only those with FDR < 0.05 were considered significant. (c) To provide a better visualization of the relationship between cardiometabolic measures with impulsivity, and only for those cardiometabolic regressors that were statistically significant in the multiple regression analysis, we defined two groups: participants with lower (≤ percentile 50th measure of interest) and higher (> percentile 50th measure of interest) cardiometabolic values. Then, the Mann–Whitney *U* test was used to analyze between-group differences in impulsivity test medians, and their ratio was calculated.

**Table 1:** Demographic, anthropometric, cardiometabolic and impulsivity measures in the normal-weight (NW) and overweight/obesity (OW/OB) groups

	NW (*n* = 43)			OW/OB (*n* = 65)			Test statistic	Effect size	*P*
	Mean (SD)	Range	NA	Mean (SD)	Range	NA			
*Demographic measures*									
Females (*n*)	23	–	–	30	–	–	*x*^2^ = 0.3^a^		0.58
Age (years old)	15.28 (2.08)	12–19	–	14.83 (1.99)	11–19	–	*W* = 1539^b^	*r* = 0.09^N^	0.37
*Anthropometric measures*									
BMI *z*-score	− 0.06 (0.59)	− 1.3 to 0.9	–	1.95 (0.38)	0.8–2.69	–	*W* = 2.5^b^	*r* = 0.84^L^	< 0.01*
WC (cm)	69.85 (7.2)	59–90	–	95.08 (10.24)	70–117	1	*W* = 75.5^b^	*r* = 0.79^L^	< 0.01*
*Cardiometabolic measures*									
SBP (mmHg)	107.78 (12.3)	82.5–132	–	116.69 (9.84)	96–135	–	*W* = 813.5^b^	*r* = 0.35^M^	< 0.01*
DBP (mmHg)	60.32 (7.95)	45–82.5	–	67.95 (8.92)	50–85	–	*W* = 727.5^b^	*r* = 0.41^M^	< 0.01*
Glucose (mmol/L)	4.32 (0.4)	3.12–4.91	–	4.52 (0.41)	3.62–5.39	–	*W* = 1043^b^	*r* = 0.21^S^	0.03*
HbA1c (%)	5.06 (0.24)	4.4–5.5	–	5.19 (0.28)	4.5–5.7	1	*t* = − 2.48^c^	*d* = − 0.49^S^	0.015*
TC (mmol/L)	4.06 (0.6)	2.9–5.95	–	3.9 (0.62)	2.67–5.58	–	*t* = 1.3^c^	*d* = 0.25^S^	0.2
HDL-c (mmol/L)	1.53 (0.32)	1.05–2.35	–	1.22 (0.25)	0.67–1.76	–	*t* = 5.69^c^	*d* = 1.12^L^	< 0.01*
LDL-c (mmol/L)	2.21 (0.49)	1.3–3.5	–	2.25 (0.54)	1.3–3.5	–	*t* = − 0.38^c^	*d* = − 0.07^N^	0.7
TG (mmol/L)	0.65 (0.25)	0.34–1.63	–	0.96 (0.44)	0.35–2.02	–	*t* = − 4.51^c^	*d* = − 0.82^L^	< 0.01*
*Estimated intelligence quotient*									
WAIS-III/WISC-IV vocabulary	11.07 (2.43)	8–19	–	10.31 (2.17)	7–17	*–*	*t* = 1.7	*d* = 0.33^S^	0.09
*Impulsivity measures*									
TFEQ-R18 Emotional eating	4.58 (2.01)	3–11	–	4.86 (1.83)	3–9	*–*	*W* = 1235^b^	*r* = 0.1^S^	0.29
TFEQ-R18 Uncontrolled eating	16.37 (5.13)	3–29	–	17.03 (5.22)	9–30	*–*	*W* = 1306^b^	*r* = 0.060^N^	0.57
TCI-R Novelty seeking total score	104.49 (11.25)	82–129	6	103.44 (11.69)	79–130	15	*t* = 0.42^c^	*d* = 0.09^N^	0.67
CPT-II Commission errors	19.5 (6.39)	3–30	1	23.45 (7.13)	5–35	–	*t* = − 2.9^c^	*d* = − 0.58^M^	< 0.01*
Stroop Interference score	3.49 (5.7)	− 9.19 to 20.37	–	2.77 (6.86)	− 16.91 to 21.24	–	*t* = 0.57^c^	*d* = 0.11^N^	0.56
WCST Perseverative errors	16.48 (11.92)	4–51	1	18.66 (12.63)	5–65	–	*t* = − 1.27^c^	*d* = − 0.18^N^	0.2
DDT Geometric mean	0.01 (0.01)	0.0001–0.047	2	0.01 (0.02)	0.0001–0.117	11	*W* = 1240^b^	*r* = 0.1^S^	0.32

BMI: body mass index; WC: waist circumference; SBP: systolic blood pressure, DBP: diastolic blood pressure; HbA1c: glycated hemoglobin; TC: total cholesterol; HDL-c: high-density lipoprotein cholesterol; LDL-c: low-density lipoprotein cholesterol; TG: triglycerides, WAIS-III: Weschler Adults Intelligence Scale-III; WISC-IV: Weschler Intelligence Scale for Children-IV; TFEQ-R18: Three-Factor Eating Questionnaire-R18; TCI-R: Temperament Character Inventory Revised; CPT-II: Conner’s Continuous Performance Test-II; Stroop: Stroop Color and Word Test; WCST: Wisconsin Card Sorting Test; DDT: Kirby Delay Discounting Task

Test statistics: ^a^Chi Squared Test; ^b^Mann–Whitney *U* test; ^c^*t*-test

Effect size interpretation: ^N^negligible; ^S^small; ^M^medium; ^L^large

*Significant differences between groups

**Table 2:** Multiple regression coefficients of the TFEQ-R18 emotional eating and CPT-II commission errors models

	TFEQ-R18—emotional eating			CPT-II—commission errors		
	*b*	95% CI	*P*	*b*	95% CI	*P*
Sex (female)	1.15	(0.45, 1.85)	0.002*	− 1.6	(− 4.14, 0.94)	0.22
Age (years old)	0.18	(− 0.006, 0.36)	0.06	− 0.92	(− 1.59, − 0.25)	0.009*
BMI *z*-score	0.07	(− 0.31, 0.45)	0.7	2.1	(0.71, 3.48)	0.004*
Estimated IQ (WAIS-III/WISC-IV vocabulary)	− 0.07	(− 0.23, 0.09)	0.37	− 0.11	(− 0.68, 0.45)	0.71
HDL (mmol/L)	0.33	(− 0.96, 1.63)	0.62	− 0.18	(− 4.88, 4.53)	0.94
LDL-c (mmol/L)	− 0.41	(− 1.08, 0.26)	0.23	− 0.26	(− 2.68, 2.16)	0.83
TG (mmol/L) (Log)	− 0.07	(− 0.99, 0.85)	0.88	4.09	(0.74, 7.43)	0.018*
Hb1Ac (%)	− 0.64	(− 1.94, 0.66)	0.33	0.8	(− 3.91, 5.51)	0.74
Glucose (mmol/L)	1.11	(0.26, 1.96)	0.016*	− 1.56	(− 4.66, 1.54)	0.32
DBP (mmHg)	0.06	(0.007, 0.11)	0.02*	− 0.17	(− 0.36, 0.02)	0.08
SBP (mmHg)	− 0.03	(− 0.07, 0.01)	0.23	− 0.05	(− 0.2, 0.1)	0.48

BMI: body mass index; IQ: intelligence quotient; WAIS-III: Weschler Adults Intelligence Scale-III; WISC-IV: Weschler Intelligence Scale for Children-IV; TFEQ-R18: Three-Factor Eating Questionnaire-R18; CPT-II: Conner’s Continuous Performance Test-II; HDL-c: high-density lipoprotein cholesterol; LDL-c: low-density lipoprotein cholesterol; TG: triglycerides; HbA1c: glycated hemoglobin; DBP: diastolic blood pressure; SBP: systolic blood pressure

*Significant associations (*P* values < 0.05) for the CPT and TFEQ-R18 emotional eating regression models with FDR < 0.05

## Results

Groups were not significantly different for sex, age, bulimia, anxiety, and depression symptoms (*P* > 0.05). As expected, the overweight/obesity group had a higher BMI *z*-score and waist circumference (mean = 1.95 and 95.08, respectively; *P* < 0.01) than their peers with normal weight (mean = − 0.06 and 69.85, respectively). Significant between-group differences were also found in the lipid profile: the overweight/obesity group had lower HDL-c values (*P* < 0.01) and higher triglycerides (*P* < 0.01) than the normal-weight group. Neither LDL-c (*P* = 0.7) nor total cholesterol (*P* = 0.2) were significantly different. Differences in demographic, anthropometric and cardiometabolic measures are detailed in Table 1. Regarding impulsivity measures, participants belonging to the overweight/obesity group performed higher commission errors in the CPT-II test (*P* < 0.01). No significant differences between groups were found in the other impulsivity measures (*P* > 0.05). Table 1 provides a summary of impulsivity measures for both groups.

### Cardiometabolic and impulsivity measures

After FDR correction, two out of the seven impulsivity models remained statistically significant: CPT-II commission errors [*R*^2^ adj = 0.24; *R*^2^ adj 95% CI = (0.11, 0.36), FDR = 0.0016] and TFEQ-R18 emotional eating [*R*^2^ adj = 0.18; *R*^2^ adj 95% CI = (0.06, 0.29); FDR = 0.012] models. The CPT-II model showed that for a 1% increase in triglycerides there was an increment of 0.04 commission errors in CPT-II (*P* = 0.018), and that for each unit of BMI *z*-score there was an increment of 2.1 commission errors (*P* = 0.004). Also, the TFEQ-R18 emotional eating model indicated that for each unit of glucose (mmol/L) and DBP (mmHg) there was an increment of 1.11 (*P* = 0.016) and 0.06 (*P* = 0.02) in this scale score, respectively. Table 2 provides a summary of the significant models. Correlations between cardiometabolic and impulsivity measures are included in Supplementary Material (Table S3).

**Fig. 1:**
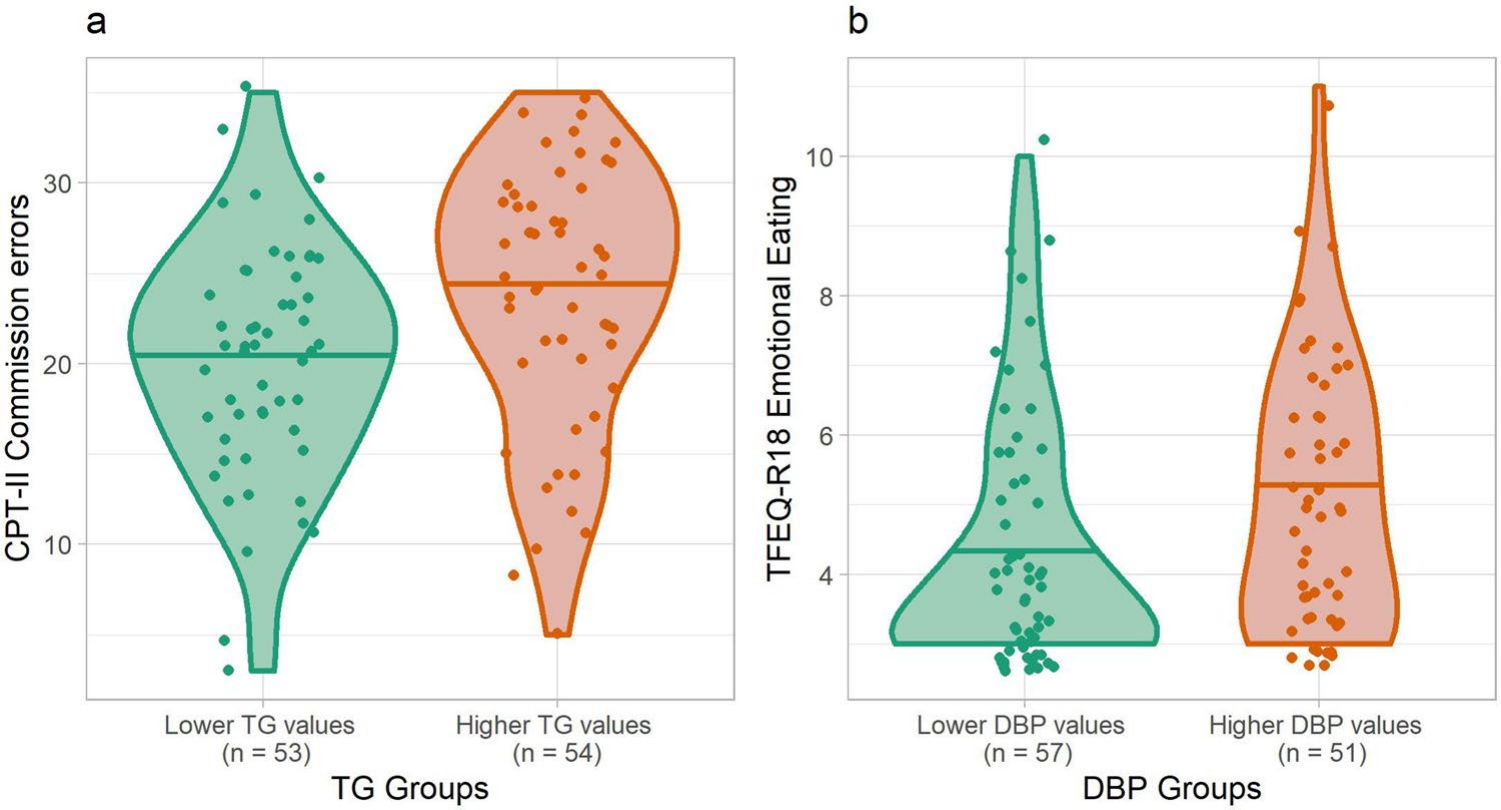
**a** Boxplot of CPT-II commission errors test (raw scores). **b** Boxplot of the TFEQ-R18 emotional eating scale (raw scores). Participants with lower TG and DBP (values ≤ median) are grouped as ‘Lower TG or lower DBP values’, whereas those with higher TG and DBP (values > median) are grouped as ‘Higher TG or higher DBP values’. Participants are not stratified by normal-weight and overweight/obesity groups. Abbreviations: CPT-II: Conners’ Continuous Performance Test-II; TFEQ-R18: Three-Factor Eating Questionnaire-R18; TG: triglycerides; DBP: diastolic blood pressure

Additionally, we tested for differences between participants with lower (values ≤ percentile 50th) and higher (values > percentile 50th) cardiometabolic values and impulsivity (Fig. 1). Participants from the higher triglycerides group (26% normal-weight, 74% overweight/obesity) committed 19% more CPT-II commission errors than those within the lower triglycerides group (53.7% normal-weight, 46.3% overweight/obesity): median = 25 and 21, respectively; *P* = 0.006. Also, participants from the higher DBP group (15.7% normal-weight, 84.3% overweight-obesity) scored 25% higher in the TFEQ-R18 emotional eating scale than those within the lower DBP group (61.4% normal-weight, 38.6% overweight/obesity): median = 5 and 4, respectively; *P* = 0.04. The rest of the regressors did not show significant statistical differences between higher and lower values.

### Cardiometabolic and neuroanatomical measures

**Fig. 2:**
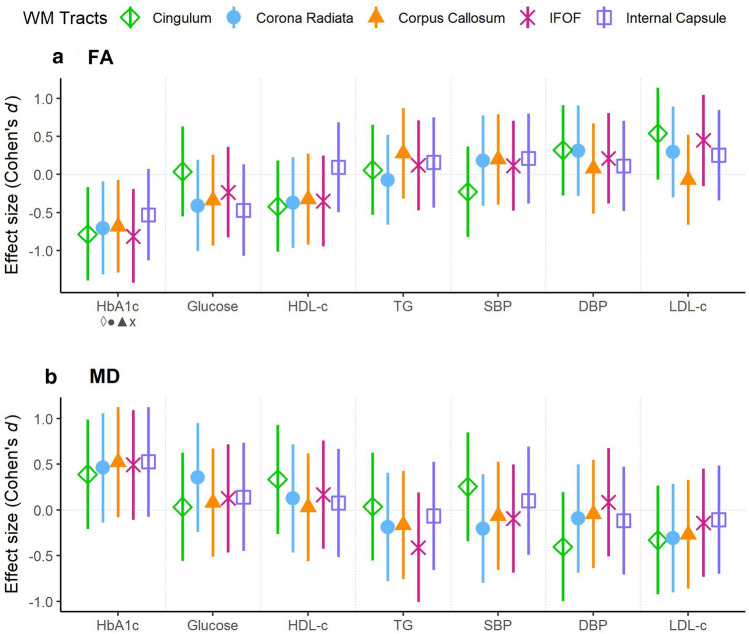
**a** Fractional anisotropy (FA), **b** mean diffusivity (MD) effect sizes—Cohen’s *d*—with 95% confidence intervals of lipid and cardiometabolic traits on the cingulum, corona radiata, corpus callosum, inferior fronto-occipital fasciculus (IFOF) and internal capsule. Significant effect sizes are labeled in the *X* axis with rhombus, circle, triangle, cross and square symbols for cingulum, corona radiata, corpus callosum, IFOF and internal capsule, respectively. After FDR correction, only the FA cingulum model remained significant. Abbreviations: DBP: diastolic blood pressure; HbA1c: glycated hemoglobin; HDL-c: high-density lipoprotein cholesterol; LDL-c: low-density lipoprotein cholesterol; SBP: systolic blood pressure; TG: triglycerides

Global FA and MD were not significantly different between groups (*P* > 0.05). In the ROI-based analysis, and regarding FA, the cingulum was significantly predicted by cardiometabolic variables [*R*^2^ adj = 0.31; *R*^2^ adj 95% CI = (0.15, 0.46); FDR = 0.015] (Table 3; Fig. 2; Supplementary Fig. S1). Specifically, glycated hemoglobin and BMI *z*-score were negatively associated with FA (*b* = − 0.01, *P* = 0.012; and *b* = − 0.002, *P* = 0.0495, respectively). Another WM tract, the IFOF, was significantly associated with glycated hemoglobin (*b* = − 0.003, *P* = 0.01), although the model did not overcome FDR correction (FDR = 0.059). Regarding MD, no significant associations were found. Correlations between cardiometabolic and neuroanatomical measures are included in Supplementary Material (Table S4). To second-assess the results obtained in the ROI-based analysis, we did a whole-brain analysis. After applying Bonferroni adjusted threshold for 32 tests (*P* < 0.0015), no significant associations were found.

**Table 3:** Multiple regression coefficients of the significant white matter tract

	FA cingulum		
	*b*	95% CI	*P*
Sex (female)	9.56e−04	(− 5.4e−03, 7.3e−03)	0.77
Age (years old)	1.84e−03	(5.45e−04, 3.1e−03)	0.008*
BMI *z*-score	− 2.35e−03	(− 4.6e−03, − 7e−05)	0.0495*
HDL (mmol/L)	− 6.53e−03	(− 1.6e−02, 2.7e−03)	0.17
LDL-c (mmol/L)	4.13e−03	(− 4.1e−04, 8.7e−03)	0.08
TG (mmol/L) (Log)	5.99e−04	(− 5.5e−03, 6.7e−03)	0.85
Hb1Ac (%)	− 1.05e−02	(− 1.8e−02, − 2.6e−03)	0.012*
Glucose (mmol/L)	3.92e−04	(− 5.6e−03, 6.3e−03)	0.9
DBP (mmHg)	2.22e−04	(− 1.9e−04, 6.3e−04)	0.3
SBP (mmHg)	− 1.3e−04	(− 4.7e−04, 2.1e−04)	0.45
Total ICV (mm^3^)	1.83e−10	(4e−10, 1e−11)	0.04*

FA: fractional anisotropy; BMI: body mass index; HDL-c: high-density lipoprotein cholesterol; LDL-c: low-density lipoprotein cholesterol; TG: triglycerides; HbA1c: glycated hemoglobin; DBP: diastolic blood pressure; SBP: systolic blood pressure; Total ICV: estimated total intracranial volume

*Significant associations (*P* values < 0.05) for the WM tract regression model with FDR < 0.05

## Discussion

We examined the relationship of cardiometabolic measures with impulsive behaviors and WM differences in adolescents with normal-weight and overweight/obesity. First, we explored the relationship that cardiometabolic measures might have with impulsivity. We found that triglycerides were associated with a more impulsive performance in the CPT-II test (higher commission errors), and that glucose and DBP were associated with higher scores on the TFEQ-R18 emotional eating scale. Second, we assessed whether cardiometabolic variables were related to WM microstructure in five ROIs. We found that FA values in the cingulum were negatively associated with glycated hemoglobin.

### Impulsivity

The most common ways to evaluate impulsivity are through rating scales and performance-based tests. Rating scales measure self-reported features of impulsive behavior over time, whereas performance-based tests provide an objective assessment of behaviors related to impulsive actions (Emery and Levine 2017). Impulsivity has been conceptualized as a broad trait composed of different phenotypes that manifest in a similar manner (Sharma et al. 2014). Within our sample, higher triglycerides were related to greater commission errors in the CPT-II test, which is also the only impulsivity measure (Shaked et al. 2020) significantly different between groups in the *t*-tests. Performance-based tests can never assess an isolated cognitive domain. It is possible that, compared to other tests, the CPT-II evaluates more directly impulsivity because it leads to more automatic responses and the capacity of inhibition becomes fundamental.

To date, there is a lacking consensus about the relationship between impulsivity and cardiometabolic measures. A study in a large healthy sample (Sutin et al. 2010) evaluated the relationship of personality traits (NEO Personality Inventory) with lipid profile. They found that impulsivity was positively associated with triglycerides, while self-discipline and deliberation were negatively associated with triglycerides and positively with HDL-c. Excitement-seeking was not significantly associated with lipid profile. Conversely, another study (Peterfalvi et al. 2019) did not find any relationship between the lipid profile (total cholesterol, HDL-c, LDL-c, triglycerides) and any CPT-II parameter in adults with major depression disorder, whereas lower HDL-c values did predict poorer shifting (WCST) abilities in this population. Although, as mentioned, there is a disparity in the literature, our results agree with previous studies that reported associations between cardiometabolic risk factors and impulsivity (Pozzi et al. 2003; Sutin et al. 2010). However, more research in clinical and healthy populations is needed to assess the nature of this relationship.

In addition, higher glucose and DBP values were related to higher scores on the TFEQ-R18 emotional eating scale. Emotional eating leads to the consumption of highly palatable and energy-dense foods—comfort foods—as a mechanism to cope with negative emotions. Given this, we hypothesize that such an eating pattern is accompanied by immediate glucose spikes and, at a mid/long term, with higher basal glucose levels. Also, negative emotions as a form of stress may be related to higher blood pressure. Particularly, the obesogenic environment and the easy access to palatable foods may be a key factor in this eating pattern, and future research studying its possible mediator effect may help to target specific public health actions.

Overall, our results support our first hypothesis. In our data, cardiometabolic risk factors are associated with impulsivity. Importantly, this association was found with cardiometabolic variables of different nature: blood pressure, glucose, and triglycerides.

### White matter microstructure

The present study provides new evidence regarding WM microstructure and cardiometabolic measures in adolescents with and without excess weight. Our results suggested an inverse association between glycated hemoglobin and FA values in the cingulum; a WM tract that has been previously related to obesity (Verstynen et al. 2012; Kullmann et al. 2015; Papageorgiou et al. 2017; Carbine et al. 2019). This finding is consistent with a recent study in healthy adults (Repple et al. 2021) that demonstrated that non-pathological variations in glycated hemoglobin are related to WM microstructure. Regarding our second hypothesis, we expected to find more cardiometabolic components associated with WM microstructure. It is possible that glycated hemoglobin, even at levels much below the prediabetes, works as an early indicator of cardiometabolic risk (Veeranna et al. 2011), whereas more morbid levels may be required for the other cardiometabolic components to show an association with WM microstructure. Also, and since our research targets adolescence—a period where individuals undergo several developmental processes, including WM maturation (Barnea-Goraly et al. 2005)—future longitudinal studies are necessary to see if our findings are related to brain maturation and myelination processes that occur in adolescence.

### Limitations and future directions

This study has some limitations that should be acknowledged: (1) given our cross-sectional design, we could not assess causality in our results, (2) the smaller sample size used for the neuroimaging analyses limited their statistical power, and (3) the diffusion-weighted images were acquired with only 30 directions. Future studies including larger sample sizes and a longitudinal approach are needed to confirm whether our findings are consistent in different age spectrums and persist over time.

## Conclusions

Our findings show that, in adolescents, triglycerides and having a BMI indicative of overweight/obesity predict a more impulsive performance in the CPT-II test (higher commission errors). In addition, glucose and DBP predict increments in the TFEQ-R18 emotional eating scale. Neuroanatomically, the cingulum FA shows a negative association with glycated hemoglobin and BMI. Our study provides a comprehensive overview of the relationship between cardiometabolic risk factors typically related to overweight/obesity and neurocognitive variables; and invites us to look beyond the BMI when evaluating possible behavioral, cognitive, and neuroanatomical differences associated with overweight/obesity.

## Supplementary Information

Below is the link to the electronic supplementary material.

Supplementary file 1 (DOCX 100 KB)

## Data Availability

The data that support the findings of this study are available from the corresponding author upon reasonable request.

## Abbreviations

BMI: Body mass index
CPT-II: Conners’ Continuous Performance Test-II
DBP: Diastolic blood pressure
DDT: Kirby Delay Discounting Task
DTI: Diffusion tensor imaging
FA: Fractional anisotropy
FDR: False discovery rate
HDL-c: High-density lipoprotein cholesterol
IFOF: Inferior fronto-occipital fasciculus
LDL-c: Low-density lipoprotein cholesterol
MD: Mean diffusivity
MRI: Magnetic resonance imaging
ROI: Region of interest
TCI-R: Temperament Character Inventory Revised
TFEQ-R18: Three-Factor Eating Questionnaire-R18
WCST: Wisconsin Card Sorting Test
WM: White matter
