# Supplementary material for: Beyond BMI: cardiometabolic measures as predictors of impulsivity and white matter changes in adolescents
Source: Brain Struct Funct. 2023 Feb 13;228(3-4):751–60. doi: 10.1007/s00429-023-02615-0 (PMC10147758; doi:10.1007/s00429-023-02615-0)
Supplement: Supplementary file 1 — (DOCX 100 KB) [file 429_2023_2615_MOESM1_ESM.docx]

**SUPPLEMENTARY MATERIAL – Brain Structure and Function**

**Beyond BMI: cardiometabolic measures as predictors of impulsivity and white matter changes in adolescents**

Anna Prunell-Castañé^a,b,c^, María Ángeles Jurado^a,b,c*^, Jonatan Ottino-González^d^, Xavier Prats-Soteras^a,b^, Consuelo Sánchez-Garre^e^, Neus Cano Marco^f^, Paloma Salas Gómez-Pablos^g^, Isabel García-García^a,b^, Maite Garolera^f,h^.

^a^ Department of Clinical Psychology and Psychobiology, University of Barcelona, Barcelona, Spain

^b^ Institute of Neurosciences, Universitat de Barcelona, Barcelona, Spain

^c^ Sant Joan de Déu Research Foundation, Barcelona, Spain

^d^ Department of Endocrinology, Children’s Hospital Los Angeles, Los Angeles, United States

^e^ Pediatric Endocrinology Unit, Hospital de Terrassa, Consorci Sanitari de Terrassa, Barcelona, Spain

^f^ Brain, Cognition and Behavior: Clinical Research, Consorci Sanitari de Terrassa, Barcelona, Spain

^g^ Catlab, Consorci Sanitari de Terrassa, Barcelona, Spain

^h^ Neuropsychology Unit, Hospital de Terrassa, Consorci Sanitari de Terrassa, Barcelona, Spain

majurado@ub.edu

**1a. Biological roles of the cardiometabolic measures**

*Cholesterol* is (i) a key component of cell membranes, (ii) needed to produce hormones (including sex hormones) and vitamin D, and (iii) needed to produce bile acids to digest food. When the level of cholesterol is above the normal range, it can combine with other substances and build up plaques in the arteries. If this situation persists, a condition known as atherosclerosis is likely to appear, which increases the cardiovascular risk. *LDL-cholesterol* contributes to plaque formation, and *HDL-cholesterol* collects excessive blood cholesterol levels and transports them to the liver for excretion. *Triglyceride*s are fundamentally involved in energy storage. Excessive levels of triglycerides contribute to atherosclerosis development. *Glucose i*s the body’s main source of energy. When eating, glucose levels spike, and the pancreas produces insulin to regulate blood glucose levels. If glucose levels are persistently high, the risk of insulin resistance and diabetes increases. The excess of blood glucose is attached to hemoglobin –*glycated hemoglobin* –, which is one of the diabetes diagnostic biomarkers. *Blood pressure* allows blood circulation. When the levels of blood pressure are high, cardiovascular risk increases (Wishart et al. 2022).

**1b. Description of impulsivity measures**

*Temperament Character Inventory Revised (TCI-R)* (Gutierrez and Bayón 2004) is a 240 questions self-administered questionnaire designed for the evaluation of the seven dimensions of personality: novelty seeking, harm avoidance, reward dependence, persistence, self-directedness, cooperativeness and self-transcendence. Novelty seeking subscale comprises a punctuation range from 35 to 175, where higher scores indicate greater impulsivity traits.

*Three-Factor Eating Questionnaire-R18 (TFEQ-R18)* (Stunkard and Messick 1985) examines cognitive and behavioral components of eating. It is an 18 self-report questionnaire that targets three eating behaviors: uncontrolled eating, emotional eating and cognitive restraint eating. Impulsiveness is assessed through emotional eating (tendency to eat in response to negative emotions) and uncontrolled eating (tendency to overeat while experiencing feelings of being out of control). Uncontrolled eating comprises a punctuation range from 9 to 36, and emotional eating from 3 to 12. In both cases, higher scores indicate greater impulsivity traits (Aoun et al. 2019)

*The Conners’ Continuous Performance Test-II (CPT-II)* (Conners 2004) is a computerized task that assesses inattention and the response inhibition component of executive function. Participants are required, with a previous short training, to press a computer key after every letter except the *X*. Commission errors are computed as the number of responses given to the non-target letter. Higher commission errors are indicative of greater impulsivity.

*The Stroop Color and Word Test* (Golden 1995) assesses the ability to inhibit cognitive interference. The Stroop test consists of three sheets with 20 words distributed in five columns each. Participants have forty-five seconds to read aloud and as fast as possible each sheet. The word-sheet (W) requires the participant to read a list of black-inked color names (i.e., red, green, blue). In the color-sheet (C), the subject is required to name the color (i.e., red, green or blue) of non-readable stimuli (i.e., “XXXX”). The incongruent-sheet (I) requires the participant to name the color of the word, which differs from the written name (i.e., “blue” in red-ink). The interference score is calculated with the formula: I – [(W x C)/W + C)]. Lower interference values denote less ability to suppress automatic responses, and thus greater impulsivity.

*The Wisconsin Card Sorting Test (WCST)* (Heaton 1999) is a computerized task that measures cognitive flexibility. Participants are asked to match 64 cards based on a principle (i.e., color, shape or number of elements) that is not explained to them and needs to be learned from the feedback as to whether their responses are correct or incorrect. After ten consecutive hits, the matching rule changes without announcement. Perseverative errors are computed as the number of incorrect responses that would have been correct for the preceding matching rule. Higher perseverative errors scores mirror impulsivity traits and cognitive rigidity, or the inability to switch from the original mindset to an alternative one.

*The Kirby Delay Discounting Task (DDT)* (Kirby 1996) is a task that measures impulsive decision-making, evaluating the preference for smaller and immediate rewards over larger and delayed rewards. Participants are asked to answer 27 questions that require them to choose between small ($25-35), medium ($50-60) and large ($75-85) delayed rewards. For example, ‘*Would you prefer $33 today or $80 in 14 days*?’. The discount rate *k* is estimated within each range (small, medium, large), and the geometric mean of the three rates is calculated for each participant. Higher k values indicate the preference for small immediate rewards, which correspond to higher levels of impulsivity.

**1c. Parameters used to acquire the diffusion weighted images**

The diffusion-weighted images (DWI) were acquired with the following parameters: repetition time = 7,700 ms, echo time = 89 ms, acquisition matrix = 122 × 122, 2 mm isotropic voxel, field of view = 244 × 244 mm2, diffusion directions = 30, slice thickness = 2 mm, number of slices = 60, b-values = 0 and 1,000 s/mm2, IPAT factor = 2 and total scan time = 4:23 minutes. A T1-weighted MPRAGE 3D sequence was also acquired for registration, EPI distortion correction, and cortical grey matter morphometry analysis using the following parameters: TR = 2300 ms, TE = 2.98 ms, inversion time = 900 ms, 240 slices, FOV = 256 × 256 mm2, 1 mm isotropic voxel.

**1d. Explanation of the DTI imaging processing procedure**

All DWI sequences were visually inspected to detect artifacts, skull-stripped and corrected for head motion and eddy currents. Parallel skull stripping and bias-filed correction (FAST) were applied to the T1-weighted images. EPI distortions of DWI images were solved by using a constrained non-rigid registration to each participants’ T1-weighted image (Bhushan et al. 2012), which is a default step from the BS diffusion pipeline (http://brainsuite.org/processing/diffusion/). Gradient rotation after this registration was also performed to optimize tensor fitting in subsequent steps. The diffusion tensor was fitted to each voxel to generate the FA maps with a linear weighted least squares model to appropriate scale data variances (Jones et al. 2013). FA maps were non-linearly registered onto the most representative participant’s FA map to prevent anatomical misalignments (Bach et al. 2014). To limit the presence of spurious tracts, the mean FA skeleton was generated based on each participants’ FA values with a threshold > 0.25. MD was also projected onto the mean FA skeleton for complementary analysis.

**Programs used for neuroimaging and statistical analyses**

FMRIB Software Library (FSL) v.6.0.4: https://fsl.fmrib.ox.ac.uk/fsl/fslwiki/FSL

BrainSuite v.18a1: http://brainsuite.org/

Freesurfer v.6.0 recon-all pipeline: https://surfer.nmr.mgh.harvard.edu/fswiki/recon-all

R statistical package v.4.0.5: https://www.r-project.org

RStudio v.1.2.5033: https:// www.rstudio.com

**References**

Aoun C, Nassar L, Soumi S, et al (2019) The Cognitive, Behavioral, and Emotional Aspects of Eating Habits and Association With Impulsivity, Chronotype, Anxiety, and Depression: A Cross-Sectional Study. Front Behav Neurosci 13:. https://doi.org/10.3389/fnbeh.2019.00204

Bach M, Laun FB, Leemans A, et al (2014) Methodological considerations on tract-based spatial statistics (TBSS). Neuroimage 100:358–369. https://doi.org/10.1016/j.neuroimage.2014.06.021

Bhushan C, Haldar JP, Joshi AA, Leahy RM (2012) Correcting Susceptibility-Induced Distortion in Diffusion-Weighted MRI using Constrained Nonrigid Registration. Signal Inf Process Assoc Annu Summit Conf APSIPA Asia Pac

Conners KC (2004) Conners’ CPT-II continuous performance test II. MHS Multi Heath Syst. Inc.

Golden CJ (1995) STROOP, test de colores y palabras. TEA Ediciones

Gutierrez JA, Bayón C (2004) Inventario del temperamento y el carácter-revisado (TCI-R). Baremación y datos normativos en una muestra de población general. Actas españolas Psiquiatr 8:8–15

Heaton RK (1999) Wisconsin Card Sorting Test: Computer Version 4-Research Edition. Psychol. Assess. Resour.

Jones DK, Knösche TR, Turner R (2013) White matter integrity, fiber count, and other fallacies: The do’s and don’ts of diffusion MRI. Neuroimage 73:239–254. https://doi.org/10.1016/j.neuroimage.2012.06.081

Kirby KMN (1996) Delay-discounting probabilistic rewards: Rates decrease as amounts increase. Psychon Bull Rev 100–104

Stunkard AJ, Messick S (1985) The three-factor eating questionnaire to measure dietary restraint, disinhibition and hunger. J Psychosom Res 29:71–83

Wishart DS, Guo AC, Oler E, et al (2022) HMDB 5.0: The Human Metabolome Database for 2022. Nucleic Acids Res 50:D622–D631. https://doi.org/10.1093/nar/gkab1062


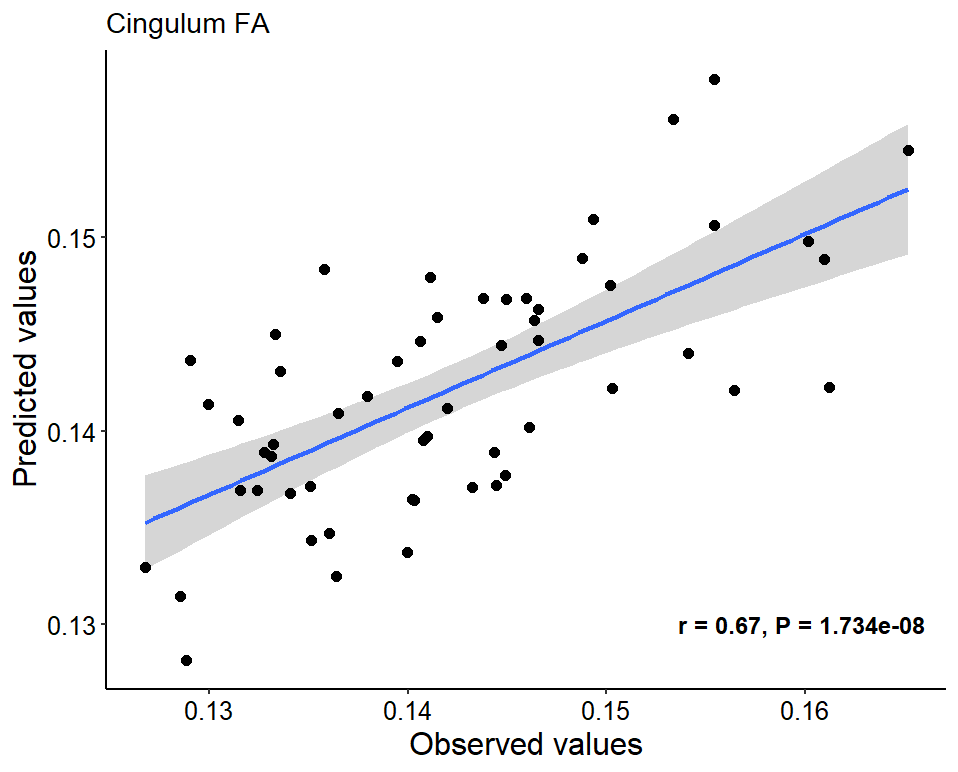


**Fig. S1** Scatterplot of the observed vs predicted cingulum FA values of the multiple regression model. All the independent variables included in the full model were used to predict cingulum FA values (sex + age + BMIz + HDL + HDL + TG(log) + Hb1Ac + glucose + DBP + SBP + Total ICV)

Table S1. Demographic, anthropometric, cardiometabolic and measures in normal-weight (NW) and overweight/obesity (OW/OB) groups of the participants that underwent an MRI acquisition.

^BMI: Body Mass Index; WC: waist circumference; SBP: systolic blood pressure, DBP: diastolic blood pressure; HbA1c: glycated hemoglobin; TC: total cholesterol; HDL-c: high-density lipoprotein cholesterol; LDL-c: low-density lipoprotein cholesterol; TG: triglycerides.^

Test statistics: ^a^ Chi Squared Test; ^b^ *t*-test; ^c^ Mann-Whitney U Test

Effect size interpretation: ^N^ negligible; ^S^ small; ^M^ medium; ^L^ large

*Significant differences between groups

Table S2. Impulsivity measures in the normal-weight (NW) and overweight/obesity (OW/OB) groups of the participants that underwent an MRI acquisition.

^TFEQ-R18: Three-Factor Eating Questionnaire-R18; TCI-R: Temperament Character Inventory Revised; CPT-II: Conner’s Continuous Performance Test-II; Stroop: Stroop Color and Word Test; WCST: Wisconsin Card Sorting Test; DDT: Kirby Delay Discounting Task.^

Test statistics: ^a^ Chi Squared Test; ^b^ *t*-test

Effect size interpretation: ^N^ negligible; ^S^ small; ^M^ medium; ^L^ large

*Significant differences between groups

Table S3. Bivariate correlations between cardiometabolic and impulsivity measures (n = 108).

Table S4. Bivariate correlations between cardiometabolic, FA and MD measures (n = 56).
